# Monthly Summary

**Published:** 1882-06

**Authors:** 


### MONTHLY SUMMARY.

*Dental Surgeons in the Army and Navy.*—A movement is on foot aiming to secure the appointment of dentists in the army and navy. It is understood that a bill for this object will be presented to Congress at its next session, and that the subject is to be brought up at the meeting of the American Medical Association in 1882.

The need of dental services in the army and navy was first urged some thirty years ago. The matter has been twice laid before Congress since then, but without any action being taken. A dentist has been appointed to the Naval Academy at Annapolis with the rank of Assistant-Surgeon, and this is, we are told, all that has been done in the matter.

The want of dentists for our soldiers and sailors is very apparent. Sound teeth are one of the physical requirements for service, and it is only right that opportunities for preserving the teeth should be given.

On sea-voyages and in Western service it is quite impossible, as things now stand, that there should be any way of treating diseased teeth except by extraction. The testimony of many of the medical staff of the army and navy, as given in the *Times* recently, is to the effect that the need of dentists is much felt.

There are a number of practical difficulties, however, in the way of creating and furnishing these services.

We have an army of only twenty thousand men scattered in small garrisons throughout the country. It would hardly be feasible to appoint a dentist for each garrison, and the dental surgeon would have, therefore, to be a rather expensive itinerant. In the navy the difficulty would be still greater. In both branches there would, no doubt, be considerable opposition to admitting dentist to equal rank with medical men. For dentist have no right to call themselves medical or surgical specialist, unless they have gone through the same kind of education and training as that to which the gynecologist, laryngologist, or oculist subjects himself.

There is no doubt, however, that dentist are often needed and would be most useful in both army and navy. We should be glad to see the obstacles in the way of the introduction of their services there overcome.—*Medical Record.*

---

*The Therapeutic uses of Nitrite of Amyl.*—Dr. Edgar Kurz, of Florence, has found his medicament so useful in the various aches and pains of every-day life that he has persuaded many families of his aquaintance to keep it on hand as a domestic remedy. It is an excellant external application for stomach-ache, colic, toothache (whether nervous or arising from caries,)

neuralgia of the trigeminus, of the cervico-brachial plexus, etc. It is superior to anything else when inhaled in so-called angio-spastic hemicrania, giving rapid relief in the individual paroxysms and prolonging the intervals betweeen them. No trial was made in case of angio paralytic hemicrania, since in this affection the drug would be physiologically contraindicated. It has a very good effect in dysmenorrhea, especially when occurring in chlorotic girls. In mild cases external applications suffice, otherwise the drug should be inhaled (when complicated with inflammatory conditions of the uterus or appendages, the results were doubtful or negative.) It was found to be of much value in attacks of dizziness and faintness occurring in anemic individuals, as also in a fainting-fit from renal colic, and in several cases of collapse during anesthesia by chloroform. It has been recommended in asphyxia from drowning, hanging, and in asphyxia of the new-born. But the first indication in these cases is the induction of artificial respiration, after the succesful initiation of which inhalations of nitrite of amyl doubtless assist in overcoming the concomitant spasm of the smaller arteries. One of the most important indications for the use of the drug is threatening paralysis of the heart from insufficient compensation. In such cases it is necessary to gain time until digitalis and alcoholics can unfold their action, and here nitrite of amyl stands pre-eminent.—*London Practitioner.*

---

*A New Fungus.*—The *Gazette Medicale* states that M. Pasteur has found an organism in the blue and green discolorations which are sometimes seen on old surgical bandages, which preserved through many cultivations the same characters and properties. It consists of colorless gloubular cells, from 1–1000 to 5-1000 of a millimeter in diameter, and has the power of secreting a pigment, which may easily be dissolved out by chloroform. The blue, or pyocyanine is reddened by acids, and restored by alkalies. Dissolved in a weak acidulated solution, neutralized by potash, and treated again with chloroform, pyocyanine yields a pure blue liquid, from which, after evaporation, it appears in the form of crystals, sometimes as prisms or matted needles, at others as rectangular plates. The watery solution of pure crystalline pyocyanine is neutral, and is unchanged by boiling.—*Medical and Surgical Reporter.*

*Therapeutic Effects of Oxygen.*—M. E. Hagen, in a report to the Academy of Sciences, gives some facts regarding the physiological and therpeutical effects of oxygen. It is taken in doses of forty to ninety litres per day, in two doses, and mixed with a very small amount of air. It augments the appetite, slightly elevates the temperature, accelerates the circulation, temporarily increases the red corpuscles and the hæmoglobim in the blood and increases the weight of the body. It stimulates the nutritive movements of the tissues, and increases thereby the excreton of the urea. In chlorosis it is a useful adjunct to iron It stand and acts much in the same way that hydrotherpy does. In vomiting it is especially valuable. After one or two inhalations vomiting will generaly stop permanently, if it be not due to organtic disease. Vomiting is relieved by oxygen when due to painful dyspepsia, dyspepsia with dilatation, vomiting of pregnancy and uræmia.—*Cincinnati Medical News.*

---

*A Triumph of Dentistry.*—At the last meeting of the Medical Society at Strasburg, reported in the *Medical Gazette* of Strasburg, Dr. Julius Böckel presented, in the name of M. Sauval, dentist, a lady for whom the latter had extracted a small molar tooth for dental caries with violent pain; and, having found it slightly carious to the bottom of its root, he sawed off the points of the root, filled it with gold carefully through the carious channel, and then re-implanted the tooth. The lady was free from all her pain; the tooth re-established itself solidly in her mouth; and at the date at which she appeared at the society (three weeks after the operation,) the tooth served for mastication as well as her other teeth. This is certainly a remarkable example of what is technically described as dental autoprothesis with aurification.—*British Medical Journal.*

---

*College Examination for the Degree of Doctorate.*—Dr. J. G. Meachem, of Racine, Wis., thinks that independent examining boards for graduates are not needed, and that the medical faculties of the several leading colleges are the best judges of the qualifications of candidates.—*Medical Record.*

